# The sequence and *de novo* assembly of *Oxygymnocypris stewartii* genome

**DOI:** 10.1038/sdata.2019.9

**Published:** 2019-02-05

**Authors:** Hai-Ping Liu, Shi-Jun Xiao, Nan Wu, Di Wang, Yan-Chao Liu, Chao-Wei Zhou, Qi-Yong Liu, Rui-Bin Yang, Wen-Kai Jiang, Qi-Qi Liang, Wangjiu, Chi Zhang, Jun-Hua Gong, Xiao-Hui Yuan, Zhen-Bo Mou

**Affiliations:** 1 Institute of Fisheries Science, Tibet Academy of Agricultural and Animal Husbandry Sciences, Lhasa 850002, China; 2 School of Computer Science and Technology, Wuhan University of Technology, Wuhan, China; 3 Novogene Bioinformatics Institute, Beijing, China; 4 College of Fishery, Huazhong Agricultural University, Wuhan, China

**Keywords:** Conservation genomics, DNA sequencing

## Abstract

Animal genomes in the Qinghai-Tibetan Plateau provide valuable resources for scientists to understand the molecular mechanism of environmental adaptation. Tibetan fish species play essential roles in the local ecology; however, the genomic information for native fishes was still insufficient. *Oxygymnocypris stewartii*, belonging to Oxygymnocypris genus, Schizothoracinae subfamily, is a native fish in the Tibetan plateau living within the elevation from roughly 3,000 m to 4,200 m. In this report, PacBio and Illumina sequencing platform were used to generate ~385.3 Gb genomic sequencing data. A genome of about 1,849.2 Mb was obtained with a contig N50 length of 257.1 kb. More than 44.5% of the genome were identified as repetitive elements, and 46,400 protein-coding genes were annotated in the genome. The assembled genome can be used as a reference for future population genetic studies of *O. stewartii* and will improve our understanding of high altitude adaptation of fishes in the Qinghai-Tibetan Plateau.

## Background & Summary

The Qinghai-Tibetan Plateau (QTP) is the largest and highest plateau in the world^1^. The upshift of QTP has formed complex mountain systems in Southwest China and greatly reshaped the drainage at this area^2^. The rapid alteration of topography in the QTP might act as significant barriers for gene flow of many species, leading to population isolations and initiating allopatric divergence and speciation^3^. Genomes of fish species in the QTP provide valuable resources for scientists to understand the molecular mechanism of environmental adaptation. Although we have successfully obtained the reference genome of *Glyptosternon maculatum*^4^, leading to the first high-quality fish genome in Tibet-plateau, the genome information of fish species in QTP is still lacking.

The schizothoracine fishes (*Schizothoracinae* subfamily, *Cyprinidae* family, *Cypriniformes* order), also known as “mountain carps”, which composed of approximately 100 species in 10–13 genera^5^. They can be diagnosed by two lines of enlarged scales along both sides of the urogenital opening and anus^6^. These fishes exhibit many unique traits that adapt to the extreme environment of the QTP^7^. Therefore, this taxon provides an excellent opportunity for investigating high altitude adaptation of teleost fishes.

Distributed in the QTP and its surrounding areas, they are the largest and most diverse taxon of the QTP icthyofauna^6^. Based on morphological traits, the schizothoracine fishes can be divided into three hierarchical groups that adapt to different environments of QTP: the primitive group (including *Schizothorax*, *Schizocypris*, and *Aspiorhynchus*), the specialized group (including *Diptychus*, *Gymnodiptychus*, and *Ptychobarbus*), and the highly specialized group (including *Gymnocypris*, *Oxygymnocypris*, *Chuanchia*, *Herzensteinia*, *Platypharodon*, and *Schizopygopsis*)^6^. The evolution of the three groups was proposed to be associated with the upshift history of the plateau^6,8^. Thus, schizothoracine fishes represent an excellent model for the study of speciation caused by geographical isolation, as well as a good model for the study of adaptive evolutions of fish species in the QTP.

Another prominent feature in the evolution of schizothoracine fishes is the complex chromosome compositions, and the majority of fishes in this taxon are considered to be polyploids^9^. Whole genome duplication (WGD) plays a vital role in the evolutionary history of plant and animals. There are at least three rounds of whole genome duplications early in teleost diversification^10,11^, and these events were suggested to be causally related to the evolutionary success of teleost^12,13^. The polyploid nature and rapid diversification of schizothoracine fishes make them a good model for the study of polyploidy driven speciation.

**Figure 1:**
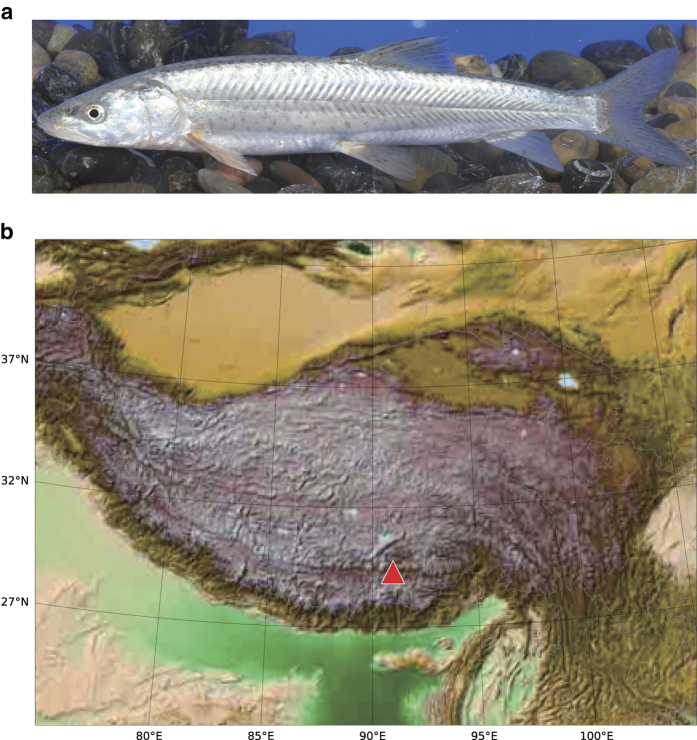
A picture of *Oxygymnocypris stewartii*. (**a**) The appearance of *Oxygymnocypris stewartii*; (**b**) Distributed localization (red triangle) of *Oxygymnocypris stewartii* for the genomic sequencing.

*Oxygymnocypris stewartii* (Lloyd, 1908) (NCBI Taxon ID: 361644, Fig. 1a), a highly specialized schzothoracine fish, is a one-time spawning fish species mainly distributed in the tributaries of the middle reaches in the YarlungZangbo River across an elevation ranging from roughly 3,000 m to 4,200 m^14^ (Fig. 1b). *O. stewartii* is currently listed in the Red List by the World Conservation Union (IUCN) and identified as an endangered fish^15^. Therefore, it is imperative to protect and restore the population resources of the *O. stewartii*.

In this report, we provide the whole genome sequence of *O. stewartii* through the PacBio single molecule sequencing technique (SMRT). The availability of a fully sequenced and annotated genome is essential to support basic biological studies and will be helpful to the development of further protection strategies for this endangered species. Its whole genome sequence will also provide a foundation to explore the adaptive evolutionary processes of highland fishes, supplied as a starting point to study speciation mechanisms caused by the rapid rising of the QTP.

## Methods

### Sample collection and sequencing

A healthy female fish captured from Gongga Country, Lhasa, Tibet (Fig. 1a,b) was used for genome sequencing. Genomic DNA was isolated using Qiagen DNA purification kit (Qiagen, Valencia, CA, USA) from the white muscular tissue as in our previous studies^4^.

To generate enough read data for the genome assembly, both the PacBio SEQUEL and the Illumina HiSeq 4000 platform were used for the sequencing. Long reads generated from the PacBio platform were used for genome assembly, and the short but accurate reads from the Illumina platform were analyzed for genome survey and base level correction after the assembly. For the PacBio platform, genomic sequencing libraries were constructed according to the PacBio suggested protocol and 141.1 Gb long sequencing reads were obtained from 27 SMRT cells. A total of 140.7 Gb (coverage of 74.3×) subreads were obtained after removing adaptors in polymerase reads (Table 1). The subreads N50 and average lengths were 14.2 and 9,0 kb, respectively. For the Illumina HiSeq 4000 sequencing platform, one ug genomic DNA molecules were used for sequencing library construction. DNA molecules were fragmented, end-paired and ligated to the adaptor, which was further fractionated on agarose gels and purified by PCR amplification. To improve the representativeness of reads for the *O. stewartii* genome, 11 paired-end sequencing libraries were constructed with insert length of 250 bp according to Illumina’s protocol (Illumina, San Diego, CA, USA). Finally, a total of 145.4 Gb (coverage of 70.8×) short sequencing reads were generated. Reads with the adaptors and a quality value lower than 20 (corresponding to a 1% error rate) were filtered out. As a result, we obtained 144.3 Gb cleaned reads for the k-mer analysis and base correction of the genome (Table 1).

The individual used for the genomic sequencing was also used for the transcriptome sequencing, providing necessary gene expression data for the genome sequence annotation. Given that gene expression exhibited clear tissue-specificity, 12 tissues, including skin, eye, swim bladder, muscle, brain, gill, heart, liver, gut, ovary, fat tissue and kidney were collected for the following transcriptome sequencing. As per the similar method in our previous study^4^, RNA molecules were extracted using RNAiso Pure RNA Isolation Kit (Takara, Japan) for all samples, and DNase I treatment was performed to eliminate DNA contamination. After the quality assessment of the extracted RNAs using NanoVue Plus spectrophotometer (GE Healthcare, NJ, USA), RNA-seq libraries were constructed according to the protocol^4^ and were sequenced by Illumina HiSeq 4000 in paired-end 150 bp mode, resulting in a total of ~50 Gb transcriptome data. All genome and transcriptome sequencing data were summarized in Table 1.

### *De novo* assembly of *Oxygymnocypris stewartii* genome

**Figure 2:**
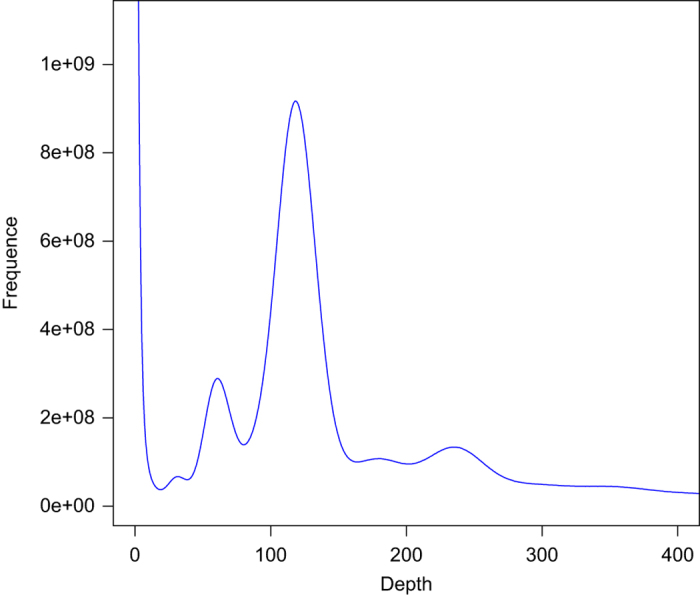
17-mer frequency distribution in *Oxygymnocypris stewartii* genomes. The X-axis is the Kmer depth, and Y-axis represents the frequency of the *K*mer for a given depth.

Genome size was estimated using Illunima sequencing data with the Kmer-based method^16^. As per our previous study^4^, we estimated the genome size of *O. stewartii* by the *K*mer frequency distribution. Jellyfish (v2.1.3)^17^ was used to calculate the frequency of each *K*mer from the short sequencing data (Table 2 and Fig. 2). As a result, we estimated the genome size of *O. stewartii* to be approximately 1,893.5 Mb.

The long reads generated from the PacBio SEQUEL platform were assembled into contigs using the FALCON package^18^ with default parameters. After the self-error correction step in the FALCON, we got 104.9 Gb (55.4x coverage) of error-corrected pre-assembly reads. The assembly of the PacBio data alone resulted in a genome of 1,898.4 Mb with a contig N50 length of 240.3 kb. The assembled genomic sequences were further polished by two rounds of polishing with Quiver^19^ using the PacBio long reads. After that, another round of the genome-wide base-level correction was performed with the Illumina short sequencing data by Pilon^20^. In the end, we obtained the final 1,849 Mb draft genome of *O. stewartii* with a contig N50 length of 257.1 kb (Table 3).

The completeness and the accuracy of the genome were evaluated by CEGMA, BUSCO and read mapping. The completeness of the genome assembly was assessed by the single copy orthologs (BUSCO, version 3.0)^21^ and CEGMA^22^ software. 94.2% complete and 3.6% partial of the 2,586 vertebrate BUSCO genes were identified in the final assembly. Using CEGMA^22^, we revealed that 95.56% of the 248 core genes were evolutionarily conserved genes identified in the genome. Both BUSCO and CEGMA confirmed the completeness of the genome assembly. The accuracy of the genome was evaluated by the Illumina short read mapping with BWA^23^ and the transcript alignment with BLAT^24^. More than 98.6% of the reads were aligned to the genome, and the insert length distribution exhibited a single peak that was consistent with the experimental design. Meanwhile, the transcriptome was *de novo* assembled by Trinity^25^, and the transcripts were mapped to the genome assembly using BLAT^24^ with default parameters. We found that the alignment coverage (alignment length to transcript length) of expressed genes ranged from 96.44 to 99.95% in the genome assembly.

### Repetitive element and non-coding gene annotation in the *O. stewartii* genome

**Figure 3:**
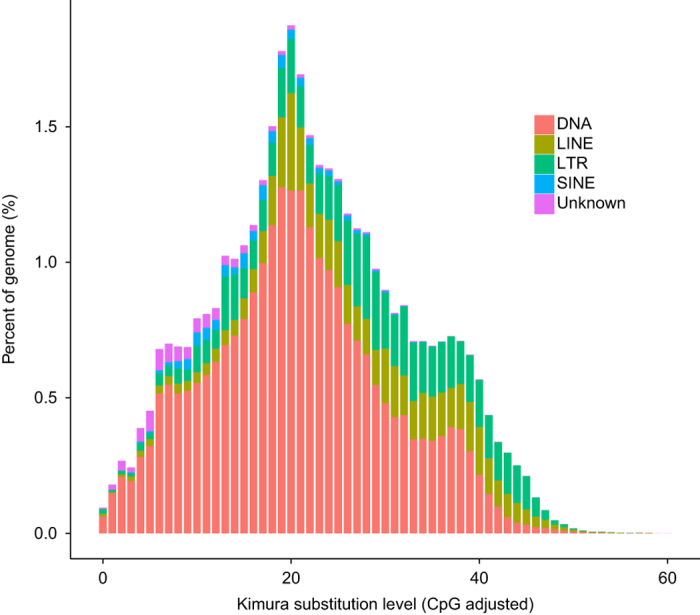
Distribution of the divergence rate of each type of repetitive element in *Oxygymnocypris stewartii* genome. The divergence rate was calculated between the identified TE elements in the genome by the homology-based method and the consensus sequence in the Repbase.

To annotate repeat elements in the *O. stewartii* genome, both homologous comparison and *ab initio* prediction were applied. The similar annotation process in our previous work^4^ was employed. For *ab initio* repeat annotation, LTR_FINDER^26^, RepeatScout^27^, and RepeatModeler (http://repeatmasker.org/RepeatModeler/) were used to construct a *de novo* repetitive element database, and the RepeatMasker^28^ (http://repeatmasker.org/RMDownload.html) were used to annotate repeat elements with the database. Then, RepeatMasker and RepeatProteinMask^28^ were used for known repeat element types by searching against Repbase database^29^. Tandem repeats were also *ab initio* predicted using TRF tool^30^. A total of 822.84 Mb repetitive elements were identified in the *O. stewartii* genome by those repeat annotation processes, accounting for 44.50% of the whole genome (Tables 4 and 5 and Fig. 3).

For non-coding genes, 24,208 tRNAs were predicted using tRNAscan-SE^31^, and 1,363 rRNA genes were annotated using BLASTN tool with an E-value of 1E-10^32^ against human rRNA sequence. Small nuclear and nucleolar RNAs in the *O. stewartii genome* were also annotated by the infernal tool^33^ using Rfam database^34^ (Table 6).

### Protein-coding gene prediction and functional annotation

**Figure 4:**
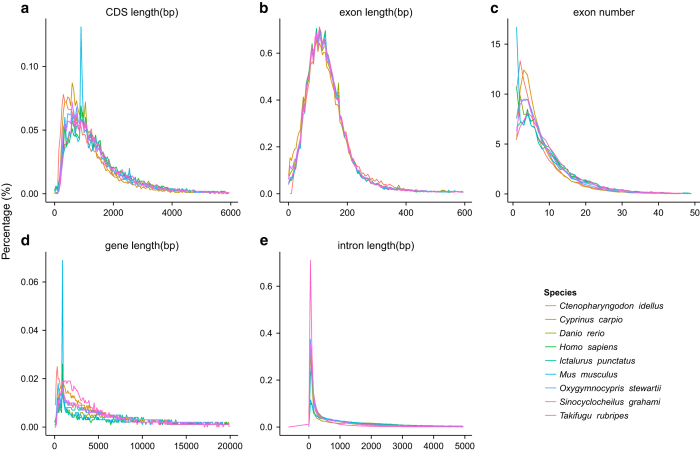
Comparisons of the prediction gene models in the *Oxygymnocypris stewartii* genome to other species. (**a**) CDS length distribution and comparison with other species. (**b**) Exon length distribution and comparison with other species. (**c**) Exon number distribution and comparison with other species. (**d**) Gene length distribution and comparison with other species. (**e**) Intron length distribution and comparison with other species.

The gene model prediction method in our previous study^4^ was applied to the protein-coding gene annotation in the *O. stewartii* genome. We merged the evidence of the gene prediction from multiple methods, including homolog based, *ab initio* and RNA-seq based annotations. The protein and coding sequences were obtained from the Ensembl database^35^ for the following species, including human (*Homo sapiens,* GCF_000001405.37), mouse (*Mus musculus,* GCF_000001635.26), zebrafish (*Barchydanio rerio var,* GCF_000002035.5), common carp (*Cyprinus carpio,* GCF_000951615.1), tiger puffer (*Takifugu rubripes,* GCF_000180615.1), channel catfish (*Ictalurus punctatus,* GCF_001660625.1), *Sinocyclocheilus graham* (GCF_001515645.1) and grass carp^36^ (*Ctenopharyngodon idellus*). The protein sequences were aligned against the *O. stewartii* genome using TBLASTN^37^ search with parameters of e-value 1e-5. After filtering low-quality records, the gene structure was predicted by GeneWise^38^ (referred to “Homology” in Table 7). Secondly, transcripts assembled from twelve tissues RNA-Seq data were aligned against the *O. stewartii* genome using Program to Assemble Spliced Alignment (PASA)^39^ (referred to “PASA” in Table 7). Augustus^40^, GeneID^41^, GeneScan^42^, GlimmerHMM^43^, and SNAP^44^ were used for *ab initio* prediction with the optimized parameters that trained using high-quality proteins that derived from the PASA gene models. RNA-seq reads were also aligned to the *O. stewartii* genome directly using TopHat^45^ v2.0.9, and the gene models were constructed by Cufflinks^46^ v2.2.1 (referred to Cufflinks in Table 7). Finally, EvidenceModeler^39^ was applied to combine all gene models that were predicted by various methods with the identical weights with our previous work^4^. Untranslated regions (UTRs) and alternative splicing variations were annotated using PASA2^39^ (referred to “PASA-update” in Table 7). Finally, 46,400 protein-coding genes with a mean of 8.41 exons per gene (Table 7) were annotated in the *O. stewartii* genome. The statistics of gene models, including lengths of a gene, CDS, intron, and exon in *O. stewartii* were comparable to those for close-related species (Table 8 and Fig. 4).

**Figure 5:**
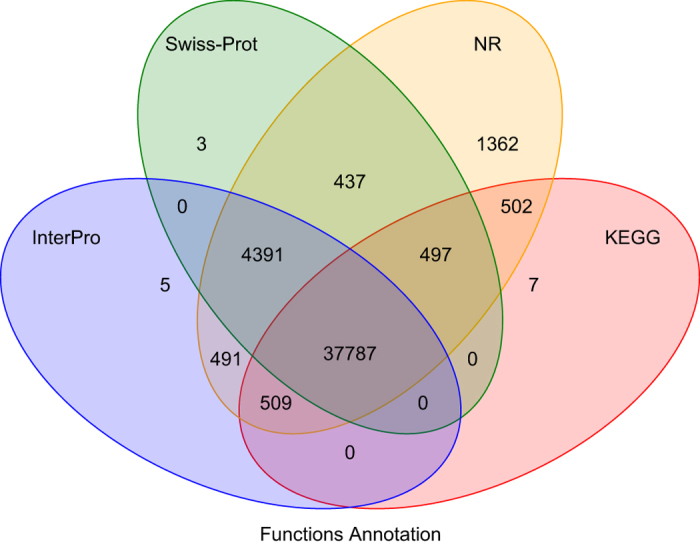
Venn diagram of the number of genes with functional annotation using multiple public databases.

Public biological function databases of SwissProt^47^, InterPro^48^, NR from NCBI and Kyoto Encyclopedia of Genes and Genomes (KEGG)^49^ were used for the functional annotation of the predicted genes. BLASTX utility^32^ were used for the homolog search with an E-value threshold of 1E-5. InterPro database^48^ was used to predict protein function based on the conserved protein domains by InterproScan tool^50^. A total of 45,991 genes (99.1%) were successfully annotated by at least one public database. (Table 9 and Fig. 5).

### Code Availability

The sequence data were generated using the software provided by the sequencing platform manufacturer and the sequencing data were processed with commands with the guidance from the public software that is cited in the manuscript. No custom computer codes were generated in this work.

## Data Records

All PacBio long-read sequencing data and Illumina short-read sequencing data have been deposited to NCBI Sequence Read Archive (SRA) (Data Citation 1).

The transcriptome data are available through the NCBI SRA (Data Citation 2).

The assembled genome version is available at GenBank (Data Citation 3).

The annotation gff3 file of the assembled genome is available at Figshare (Data Citation 4).

## Technical Validation

### RNA integrity

The transcriptomes for twelve tissues from three fish individuals were sequenced. Before constructing RNA-Seq libraries, the concentration and quality of total RNA were evaluated using NanoVue Plus spectrophotometer (GE Healthcare, NJ, USA). The total amount of RNA, RNA integrity and rRNA ratio were used to estimate the quality, content and degradation level of RNA samples. In the present study, RNAs samples with a total RNA amount ≥10 μg, RNA integrity number ≥8, and rRNA ratio ≥1.5 were finally subjected to construct the sequencing library.

### Quality filtering of Illumina sequencing raw reads

The raw sequencing reads generated from the Illumina platform were rigorously cleaned by the following procedures as in the previous study^4^. Firstly, adaptors in the reads were filtered out; secondly, reads with more than 10% of N bases were filtered out; thirdly, reads with more than 50% of the low-quality bases (phred quality score <= 5) were filtered out. If any end pair was classified as low quality, both pairs were discarded. The initially generated raw sequencing reads were also evaluated for quality distribution, GC content distribution, base composition, average quality score at each position and other metrics.

## Additional information

**How to cite this article**: Liu, H. P. *et al*. The sequence and *de novo* assembly of *Oxygymnocypris stewartii* genome. *Sci. Data*. 6:190009 https://doi.org/10.1038/sdata.2019.9 (2019).

**Publisher’s note**: Springer Nature remains neutral with regard to jurisdictional claims in published maps and institutional affiliations.

## Supplementary Material



## Figures and Tables

**Table 1:** Sequencing data used for the *Oxygymnocypris stewartii* genome assembly.

Library types	Insert size (bp)	Raw data (Gb)	Clean data (Gb)	Read length (bp)	Sequence coverage (X)
Illumina reads	250	145.4	144.3	150	76.21
Pacbio reads	20,000	141.1	140.7	13,287	74.31
RNA reads	250	98.8	94.76	150	50.04
Total	—	385.3	379.76	—	200.56
Note that the coverage was calculated using the estimated genome size from *the K*mer-based method.					

**Table 2:** Statistics of 17-mer analysis for *Oxygymnocypris stewartii* genome.

*K*mer	*K*mer number	Peak depth	Genome size（Mb）	Used bases	Used reads	Coverage (X)
17	115,523,294,760	60	1,893.51	144,295,054,200	961,967,028	76.21
Note that all 17-mer sequences were extracted from paired-end clean reads that passed quality control (QC) from Next-generation sequencing libraries, and the frequency of each 17-mer was calculated and plotted in Fig. 2.						

**Table 3:** The statistics of length and number for the de novo assembled genome of *Oxygymnocypris stewartii*.

Statistics	Length (bp)	Number
Total	1,849,224,471	26,281
Max	8,753,147	—
Number >= 2000	—	25,716
N50	257,093	1,104
N60	120,727	2,199
N70	70,409	4,248
N80	44,440	7,597
N90	29,065	12,765
Note that the length statistics of the genome assembly was based on the estimated genome size from *the K*mer-based method.		

**Table 4:** The annotation of repeated sequences in the *Oxygymnocypris stewartii* genome using TRF, RepeatMasker, and RepeatProteinMask.

Type	Repeat Size(bp)	percentage of genome (%)
TRF (Tendem Repeat Finder)	151,169,214	8.17
RepeatMasker	788,753,932	42.65
RepeatProteinMask	103,914	0.01
Total	822,841,233	44.50
Note that the total content was merged and redundancy was eliminated by each method.		

**Table 5:** Summary statistics of repeat annotation in *Oxygymnocypris stewartii*.

Type	*De novo*+Repbase							TE Proteins		Combined TEs	
	Length (bp)	% in Genome	Length (bp)	% in Genome	Length (bp)	% in Genome					
DNA	294,627,292	15.93	6,140	0.0003	294,628,980	15.93
LINE	180,661,987	9.77	54,732	0.003	180,672,396	9.77
SINE	10,828,447	0.59	0	0	10,828,447	0.59
LTR	283,995,197	15.36	43,968	0.0024	284,000,105	15.36
Satellite	35,364,895	1.91	0	0	35,364,895	1.91
Simple_repeat	37,479,121	2.03	0	0	37,479,121	2.03
Unknown	25,680,794	1.39	0	0	25,680,794	1.39
Total	788,753,932	42.65	103,914	0.0056	788,758,656	42.65
Note that *De novo* + Repbase represent the result of RepeatMasker based on Repbase, RepeatModeler, RepeatScout, and LTR_FINDER; TE proteins meant the result of RepeatProteinMask based on Repbase, and the Combined TEs refer to the combined results of *De novo + *Repbase and TE proteins.						

**Table 6:** The number of the annotated non-coding RNA in the *Oxygymnocypris stewartii* genome.

Type		Number	Average length (bp)	Total length (bp)	% of genome
miRNA		1,758	106.4	187,050	0.0101
tRNA		24,208	75.45	1,826,526	0.0988
rRNA	rRNA	1,363	123.19	167,907	0.0091
	18 S	112	294.73	33,010	0.0018
	28 S	170	210.1	35,717	0.0019
	5.8 S	19	103.42	1,965	0.0001
	5 S	1,062	91.54	97,215	0.0053
snRNA	snRNA	923	132.36	122,168	0.0066
	CD-box	221	111.13	24,560	0.0013
	HACA-box	215	143.72	30,899	0.0017
	splicing	444	129.1	57,322	0.0031

**Table 7:** The statistics of gene models of protein-coding genes annotated in the *Oxygymnocypris stewartii* genome.

Methods/Tools								Gene Number	Average length (bp)				Exons number per gene
transcript	CDS	Exon	Intron										
*Ab initio*	Augustus	101,732	7,592.54	981.37	188.14	1,568.10	5.22
	GlimmerHMM	223,822	7,337.30	534.39	154.34	2,762.67	3.46
	SNAP	198,963	10,915.28	755.07	150.73	2,534.08	5.01
	Geneid	97,442	10,811.87	1,010.54	230.95	2,903.65	4.38
	Genscan	95,641	12,679.26	1,184.24	200.27	2,339.66	5.91
Homolog	*Takifugu rubripes*	53,733	8,271.98	1,195.23	202.14	1,440.46	5.91
	*Ctenopharyngodon idellus*	70,092	6,457.54	1,162.26	217.66	1,220.15	5.34
	*Danio rerio*	63,215	8,466.61	1,261.85	206.17	1,407.08	6.12
	*Cyprinus carpio*	78,104	6,467.89	1,176.98	227.61	1,268.52	5.17
	*Mus musculus*	44,944	9,259.53	1,202.88	189.77	1,509.12	6.34
	*Ictalurus punctatus*	59,212	8,747.74	1,268.06	205.62	1,447.61	6.17
	*Sinocyclocheilus grahami*	70,380	7,956.29	1,204.04	205.73	1,391.50	5.85
	*Homo sapiens*	46,698	9,041.85	1,176.04	189.54	1,511.28	6.2
RNA-seq	Cufflinks	93,109	21,118.90	3,436.98	357.39	2,051.98	9.62
	PASA	140,045	10,537.33	1,152.91	165.15	1,569.00	6.98
EVM								101,031	8,674.09	1,018.48	183.65	1,684.16	5.55
PASA-update								100,450	8,739.14	1,026.34	184.60	1,691.47	5.56
Final set								46,400	13,348.16	1438.34	171.04	1,607.39	8.41
Note that: CDS refers to coding sequence; GlimmerHMM was a new gene finder based on a Generalized Hidden Markov Model (GHMM); SNAP refers to Semi-HMM-based Nucleic Acid Parser; EVM refers to Evidence modeler.							

**Table 8:** The comparison of the gene models annotated from the *Oxygymnocypris stewartii* genome and other teleosts.

Species	Gene Number	Average length (bp)				Exons number per gene
		transcript	CDS	Exon	Intron	
*Oxygymnocypris stewartii*	46,400	13,348.16	1438.34	171.04	1,607.39	8.41
*Ctenopharyngodon idellus*	32,811	10444.53	1384.98	180.99	1361.89	7.65
*Homo sapiens*	19,805	43772.47	1457.89	171.22	5631.04	8.51
*Mus musculus*	22,278	37435.55	1600.64	179.14	4516.11	8.93
*Sinocyclocheilus grahami*	45,899	16243.9	1585.31	171.68	1780.2	9.23
*Takifugu rubripes*	21,317	8334.84	1699.01	165.45	715.91	10.27
*Danio rerio*	25,619	25207.59	1642.64	174.39	2798.97	9.42
*Cyprinus carpio*	49,264	11780.68	1260.28	163.96	1573.34	7.69
*Ictalurus punctatus*	22,966	17866.19	1760.81	170.99	1732.19	10.3

**Table 9:** The number of genes with homology or functional classification for *Oxygymnocypris stewartii*.

Database		Annotated Num	Annotated Percent (%)
NR		45,976	99.1
Swiss-Prot		43,115	92.9
KEGG		39,302	84.7
InterPro	All	43,183	93.1
	Pfam	38,742	83.5
	GO	31,811	68.6
Annotated		45,991	99.1
Total		46,400	-
